# Undiagnosed depression, persistent depressive symptoms and seeking mental health care: analysis of immigrant and non-immigrant participants of the Canadian Longitudinal Study of Aging

**DOI:** 10.1017/S2045796020000670

**Published:** 2020-08-14

**Authors:** D. Farid, P. Li, D. Da Costa, W. Afif, J. Szabo, K. Dasgupta, E. Rahme

**Affiliations:** 1 Department of Family Medicine, McGill University, Montreal, Quebec, Canada; 2 Centre for Outcomes Research and Evaluation, Research Institute of the McGill University Health Centre, Montreal, Quebec, Canada; 3 Department of Pediatrics, McGill University, Montreal, Quebec, Canada; 4 Department of Medicine, Division of Clinical Epidemiology, McGill University, Montreal, Quebec, Canada; 5 Department of Medicine, Division of Gastroenterology, McGill University Health Center, Montreal, Quebec, Canada; 6 Chronic Viral Illnesses Service, McGill University Health Center, Montreal, Quebec, Canada; 7 Department of Medicine, Division of General Internal Medicine, McGill University, Montreal, Quebec, Canada

**Keywords:** CLSA, cohort, depression, immigrant, mental health, national representative sample

## Abstract

**Aims:**

Early diagnosis and treatment of depression are associated with better prognosis. We used baseline data of the Canadian Longitudinal Study on Aging (2012–2015; ages 45–85 years) to examine differences in prevalence and predictors of undiagnosed depression (UD) between immigrants and non-immigrants at baseline and persistent and/or emerging depressive symptoms (DS) 18 months later. At this second time point, we also examined if a mental health care professional (MHCP) had been consulted.

**Methods:**

We excluded individuals with any prior mood disorder and/or current anti-depressive medication use at baseline. UD was defined as the Center for Epidemiological Studies Depression 10 score ⩾10. DS at 18 months were defined as Kessler 10 score ⩾19. The associations of interest were examined in multivariate logistic regression models.

**Results:**

Our study included 4382 immigrants and 18 620 non-immigrants. The mean age (standard deviation) in immigrants was 63 (10.3) years *v.* 65 (10.7) years in non-immigrants and 52.1% *v.* 57.1% were male. Among immigrants, 12.2% had UD at baseline of whom 34.2% had persistent DS 18 months later *v.* 10.6% and 31.4%, respectively, among non-immigrants. Female immigrants were more likely to have UD than female non-immigrants (odds ratio 1.50, 95% confidence interval 1.25–1.80) but no difference observed for men. The risk of persistent DS and consulting an MHCP at 18 months did not differ between immigrants and non-immigrants.

**Conclusions:**

Female immigrants may particularly benefit from depression screening. Seeking mental health care in the context of DS should be encouraged.

## Introduction

Depression is associated with lower quality of life (Ishak *et al*., 2013), higher risk of suicide (Chandrasena *et al.*, 1991; Ono, 2004; Murray *et al.*, 2012; Briggs *et al*., 2018), disability and loss of productivity (Lim *et al.*, 2008; Egede, 2007; Alonso *et al*., 2011; Volkert *et al.*, 2013; Greenberg *et al.*, 2015; Patten *et al*., 2015; Evans-Lacko and Knapp, 2016; Ferenchick *et al.*, 2019). Unfortunately, depression is often undiagnosed because of failure to recognise the symptoms and/or to seek mental health care (Wang *et al.*, 2007; Collerton *et al.*, 2009; Pelletier *et al*., 2017). Delays in treatment are linked to lower remission and poorer prognosis (Licht-Strunk *et al*., 2009; Ghio *et al.*, 2015). In 2012, 50–67% of all cases of depression were undiagnosed in Canada (Pelletier *et al*., 2017).

Only around 40% of Canadians with mental health disorders seek mental health care (Vasiliadis *et al*., 2007). In particular, immigrants seem to underutilise mental health services (Chen and Kazanjian, 2005; Fenta *et al.*, 2006; Lai and Surood, 2010; Bauldry and Szaflarski, 2017; Jimenez *et al*., 2017; Yang *et al*., 2020), despite the effect of migration on their mental health well-being (Tang *et al.*, 2007; Kuo *et al*., 2008; Jafari *et al.*, 2010; Islam *et al.*, 2014; George *et al*., 2015; Guruge *et al.*, 2015*b*). Immigrants differ from non-immigrants in their behaviour towards healthcare seeking in general [predisposing (e.g. language barrier), enabling (e.g. employment, knowledge of healthcare system, community support) and needs factors (health status and perceived mental health)] (Tiwari and Wang, 2008; Straiton *et al*., 2014; Subedi and Rosenberg, 2014), as described by Andersen's behavioural model of health services use (Andersen, 1995; Babitsch *et al*., 2012). The individual's willingness to seek care varies by their cultural shaping of symptoms, belief structures and illness behaviours (Aday and Andersen, 1974; Kirmayer *et al*., 2011). Otherwise, structural barriers such as candidacy (migrants' eligibility for medical attention and intervention), lack of trust between patient and their physician, delayed diagnosis or under-referral can also diminish access to mental healthcare services in some immigrant groups (Aday and Andersen, 1974; Dixon-Woods *et al*., 2006; Freeman *et al.*, 2011; Kirmayer *et al.*, 2011).

Over 20% of the Canadian population are immigrants (Chavez, 2019). However, little is known about their risk of undiagnosed depression (UD) and about their mental health care-seeking behaviours (Ali, 2002; Kuo *et al*., 2008; Sahai-Srivastava and Zheng, 2011). These issues may be particularly salient in females who generally have higher rates of depression than males (Canadian Psychiatric Association, 2001; Patten *et al*., 2015; National Institute of Mental Health, 2017).

Some authors have examined the risk of depression in immigrants compared to non-immigrants (Foo *et al*., 2018; Islam *et al*., 2014; Kuo *et al.*, 2008; Gushulak et al., 2011), but none examined the risk of UD in this group. Reviews that examined the risk of depression in immigrants reported inconclusive results (Noh *et al*., 1992; Swinnen and Selten, 2007; Cook *et al.*, 2009; Foo *et al*., 2018; Lee, 2019). Of note, moderating effects of length of stay in the host country, age at immigration, education attainment and employment status on risk of depression were reported (Kuo *et al.*, 2008; Islam *et al*., 2014; Foo *et al*., 2018; Gushulak et al., 2011). Recently, one Canadian study found that the trajectory of deterioration in mental health for older immigrants is not linear with respect to the length of stay (Davison *et al*., 2019) and further longitudinal investigation is needed.

Among Canadians who participated in baseline data collection of the Canadian Longitudinal Study on Aging (CLSA), Comprehensive cohort in 2012–2015 (ages 45–85 years), and who had not been previously diagnosed with any mood disorder and were not using an anti-depressive agent, we assessed associations between immigration status and the presence of UD at baseline. We also evaluated the association between immigration status and the presence of depressive symptoms (DS) at 18 months in those with and those without UD at baseline. In addition, we examined the association between immigration status and consulting a mental health care professional (MHCP) at 18 months among those with and those without DS at this time point and accounting for UD at baseline.

## Method

Between 2012 and 2015, for the baseline data of its Comprehensive cohort, the CLSA recruited and collected information from community-dwelling males and females ages 45–85 years. Details about the CLSA's sampling and design have been published elsewhere (Raina *et al*., 2009). Ethics approval for the present analysis was not required by the McGill University Health Centre Research Ethics Board since the database is anonymised. We focused on the comprehensive cohort (*n* = 30 097; face-to-face interviews at baseline and computer-assisted phone interview at 18 months), excluding those with any mood disorder in the last year, current anti-depressant use, and/or missing information on the outcomes and main exposure of interest as defined below (online Supplementary Fig. 1).

Our primary outcome was UD defined by a Center for Epidemiological Studies Depression (CES-D) score ⩾10. The short form of CES-D, CES-D 10 was used in this study. This is a ten-item questionnaire with four possible choices for each question: all of the time, occasionally, some of the time, and rarely or never (Andresen *et al*., 1994). The CES-D was found to be reliable and valid to assess symptoms of depression with a cut-off score of 10 in healthy community-dwelling older adults (Andresen *et al.*, 1994; Papassotiropoulos and Heun, 1999; Vilagut *et al.*, 2016; Mohebbi *et al*., 2018). Our secondary outcomes assessed at 18 months were (1) DS measured by the Kessler Psychological Distress Scale 10 (K10) score ⩾19 (Kessler *et al*., 2003), and (2) seeking MHCP consultation for these symptoms in the prior month. K10 is a ten-item questionnaire about distress feelings with each question scored from 1 to 5 (Kessler *et al*., 2003). Individuals with a K10 ⩾ 19 were considered by several studies to be likely experiencing subclinical symptoms of depression that required medical attention to prevent worsening (Atkins *et al*., 2013; Ishak *et al.*, 2013; Honda *et al.*, 2014; Vasiliadis *et al.*, 2015; Ng *et al*., 2017). Hence, a K10 ⩾ 19 was used as a proxy for having DS. Measures of CES-D and K10 were the only depression-related measures assessed at baseline and 18 months, respectively.

Baseline characteristics were grouped into: predisposing characteristics, enabling resources, needs-related factors (health status) and personal health habits as suggested by Andersen's behavioural model (Andersen, 1995; Babitsch *et al*., 2012). Predisposing characteristics included sex, age (45–60, 61–70 and 71–85 years), immigration status (yes/no), age at immigration, time lived in Canada, marital status (widowed, divorced or separated), cultural and racial background (White, Black, South Asian, Chinese and Other) and language most spoken at home (French, English and other). Enabling resources were annual household income (Can$, <20 000; 20 000–50 000; 50 000–100 000 and >100 000), employment status (employed, unemployed and completely or partly retired), education (post-secondary, secondary, <secondary), province of residency (Ontario, British Columbia, Quebec and other) and region of residency (urban or rural/suburban) (Sherbourne and Stewart, 1991). Needs-related factors included living with pain and history of common comorbid conditions such as cancer, arthritis, bowel disorders (Crohn's disease, ulcerative colitis or irritable bowel syndrome), myocardial infarction, diabetes, hypertension and anxiety disorders (phobia, obsessive-compulsive disorders and panic disorders). Perceived health was reported in five categories ‘poor’, ‘fair’, ‘good’, ‘very good’ or ‘excellent’. Personal health choices included alcohol consumption (no, occasional or regular) in the past year; participation in social activities involving sports or physical exercise in the past year (once a day, once a week, once a month, and once a year or never); smoking status (current, former and never); and body mass index (WHO classification for adults aged ⩾18 years) (WHO, 1995).

### Statistical analysis

Descriptive statistics with means and standard deviations (s.d.) for continuous variables and counts with percentages for categorical variables were computed by immigration status. Multivariate logistic regression models were used (1) to assess the associations between immigrant status and UD; (2) to examine the association between immigrant status and DS at 18 months in those depressed and those not depressed at baseline; and (3) to examine the association between immigrant status and consulting an MHCP at 18 months among those with and without DS at this time point. Immigration status, sex, age and province were included in all models, and all models adjusted for predisposing, enabling, needs-related and health-choice factors. In the model assessing the association between immigration status and UD, we examined the interaction effect between immigration status and other predisposing, enabling and needs factors. In the model assessing the association between immigration status and DS at 18 months, we examined the interaction effect between immigration status and UD at baseline and between UD at baseline and other predisposing, enabling and needs factors. Finally, in the model assessing the association between immigration status and MHCP at 18 months, we examined the interaction effect between immigration status and DS and between DS and UD at baseline. A significance level of 0.05 and the Bayesian information criterion were used to select the final models. To make the estimates generalisable to the Canadian population and address the complexity of the CLSA survey design, we used sample weights and geographic strata information provided by the CLSA in the descriptive analyses and regression analyses (Canadian Longitudinal Study on Aging, 2017). Results were expressed in odds ratios (OR) and 95% confidence intervals (CI). The proportion of missing data was less than 5% for all variables considered except for income where it was 6.9%. Therefore, only complete data were analysed, and multiple imputations were not used. Statistical analyses were performed using SAS software package Version 9.4 (SAS Institute Inc., Cary, North Carolina, USA).

## Results

Our analyses included 23 002 individuals (online Supplementary Fig. 1). These were mostly from urban settings (87.7%; Table 1), White (95.2%) and primarily spoke English at home (81.9%). About half were men (53.0%) and most were married (71.6%). Their mean age was 63 years (s.d. 10.4 years) and over 75% had a household income above Can$ 50 000. Roughly, 85% had a post-secondary degree, over half were retired (55.9%) and 40.6% were employed. Most (65.7%) reported very good/excellent health. Hypertension (36.0%), diabetes (16.3%) and cancer (15.5%) were their most prevalent chronic diseases. One-third (32.8%) lived with pain and 7.8% had bowel disorders. Almost half consumed alcohol more than twice a week, 7.5% were current smokers, 68.6% were obese or overweight and almost half participated in a social activity involving sports or a physical exercise with others at least once a week (48.1%) (Table 1).

**Table 1.** Baseline characteristics associated with immigrant status: multivariate logistic regression models

	All respondents	Non-immigrant	Immigrant	Immigrant *v.* non-immigrant
	(*N* = 23 002)	(*N* = 18 620)	(*N* = 4382)	(*N* = 22 278)
	*N* (%)	*N* (%)		Adjusted OR (95% CI)
*Predisposing characteristics*				
Age, years, mean (s.d.)	63 (10.4)	63 (10.3)	65 (10.7)	–
45–60	9866 (42.9)	8355 (44.9)	1511 (34.5)	1
61–70	6905 (30.0)	5496 (29.5)	1409 (32.2)	1.72 (1.53–1.93)
71–85	6231 (27.1)	4769 (25.6)	1462 (33.4)	2.24 (1.96–2.57)
Sex				
Male	12 200 (53.0)	9699 (52.1)	2501 (57.1)	1
Female	10 802 (47.0)	8921 (47.9)	1881 (42.9)	0.82 (0.75–0.90)
Marital status				
Single	1808 (7.9)	1575 (8.5)	233 (5.3)	1
Married	16 476 (71.6)	13 221 (71.0)	3255 (74.3)	1.79 (1.46–2.19)
Widowed/divorced/separated	4714 (20.5)	3821(20.5)	893 (20.4)	1.38 (1.12–1.69)
Cultural and racial background^a^				
White	21 888 (95.2)	18 388 (98.8)	3500 (79.9)	–
Black	199 (0.9)	32 (0.2)	167 (3.8)	–
South Asian	255 (1.1)	8 (0.0)	247 (5.6)	–
Chinese	196 (0.9)	54 (0.3)	142 (3.2)	–
Other	399 (1.7)	75 (0.4)	324 (7.4)	–
Language most spoken at home				
French	4121 (18.1)	3830 (20.6)	291 (6.6)	1
English	18 675 (81.9)	14 768 (79.3)	3907 (89.2)	6.27 (4.88–8.05)
Length of residence in Canada (years)				
0–5	–	–	57 (1.3)	–
6–10	–	–	155 (3.5)	–
11–20	–	–	336 (7.7)	–
21–40	–	–	1075 (24.5)	–
<40	–	–	2759 (63.0)	–
Age at arrival in Canada (years)				
0–5	–	–	733 (16.7)	–
6–17	–	–	754 (17.2)	–
18–22	–	–	618 (14.1)	–
22–40	–	–	1869 (42.6)	–
<40	–	–	408 (9.3)	–
*Enabling resources*				
Total household income Can $				
<20 000	894 (4.2)	865 (4.3)	169 (3.9)	1
20 000–less than 50 000	4546 (21.2)	4023 (19.8)	943 (21.5)	0.80 (0.63–1.02)
50 000–less than 100 000	7657 (35.6)	6733 (33.1)	1479 (33.8)	0.60 (0.47–0.77)
⩾100 000	8395 (39.0)	7434 (36.6)	1452 (33.1)	0.48 (0.37–0.62)
Working status				
Employed	9317 (40.6)	7622 (40.9)	1695 (38.7)	1
Unemployed	809 (3.5)	637 (3.4)	172 (3.9)	1.27 (1.01–1.61)
Retired	12 815 (55.9)	10 311 (55.4)	2504 (57.1)	0.64 (0.57–0.72)
Education level				
<Secondary school	1204 (5.2)	1071 (5.8)	133 (3.0)	1
Secondary school	2171 (9.5)	1851 (9.9)	320 (7.3)	1.62 (1.24–2.14)
Post-secondary degree/diploma	19 589 (85.3)	15 680 (84.2)	3909 (89.2)	2.46 (1.93–3.12)
Setting				
Urban	19 918 (87.7)	16 007 (86.0)	3911 (89.3)	1
Rural/suburban	2795 (12.3)	2385 (12.8)	410 (9.4)	0.77 (0.67–0.88)
Province				
Quebec	4384 (19.1)	3856 (20.7)	528 (11.0)	1
British Columbia	4734 (20.6)	3409 (18.3)	1325 (30.2)	0.66 (0.53–0.83)
Ontario	4867 (21.1)	3713 (19.9)	1154 (26.3)	0.60 (0.48–0.74)
Other	9017 (39.2)	7642 (41.0)	1375 (31.4)	0.31 (0.25–0.39)
*Needs-related factors*				
Perceived health				
Poor	190 (0.8)	140 (0.8)	50 (1.1)	–
Fair	1324 (5.8)	1072 (5.8)	252 (5.8)	–
Good	6364 (27.7)	5068 (27.2)	1296 (29.6)	–
Very good	9966 (43.3)	8189 (44.0)	1777 (40.6)	–
Excellent	5141 (22.4)	4139 (22.2)	1002 (22.9)	–
Medical conditions (Yes *v*. No)				
Living with pain	7232 (32.8)	5846 (31.4)	1386 (31.6)	–
Bowel disorders	1798 (7.8)	1500 (8.1)	298 (6.8)	0.78 (0.66–0.92)
Arthritis	655 (2.9)	524 (2.8)	131 (3.0)	–
Myocardial infarction	1087 (4.7)	867 (4.7)	220 (5.0)	–
Stroke	351 (1.5)	274 (1.5)	77 (1.8)	–
Cancer	3551 (15.5)	2856 (15.3)	695 (15.9)	0.86 (0.77–0.97)
Hypertension	8250 (36.0)	6632 (35.6)	1618 (36.9)	–
Diabetes	3738 (16.3)	2997 (16.1)	741 (16.9)	–
Anxiety disorders	715 (3.1)	611 (3.3)	104 (2.4)	–
*Personal health choices*				
Alcohol consumption				
Never	2331 (10.4)	1845 (9.9)	486 (11.1)	–
About once a month	4095 (18.2)	3281 (17.6)	814 (18.6)	–
2–4 times a month	4865 (21.7)	4049 (21.7)	816 (18.6)	–
>2 times a week	11 155 (49.7)	9063 (48.7)	2092 (47.7)	–
Smoking status				
Smoker	1704 (7.5)	1470 (7.9)	234 (5.3)	1
Former smoker	13 697 (59.9)	11 248 (60.4)	2449 (55.9)	1.38 (1.13–1.68)
Non-smoker	7470 (32.7)	5795 (31.1)	1675 (38.2)	1.72 (1.41–2.11)
Weight classification^b^				
Underweight	157 (0.7)	124 (0.7)	33 (0.8)	0.93 (0.55–1.50)
Normal weight	7039 (30.7)	5586 (30.0)	1453 (33.2)	1
Overweight	9465 (41.3)	7630 (41.0)	1835 (41.9)	0.88 (0.80–0.97)
Obese	6261 (27.3)	5209 (28.0)	1052 (24.0)	0.73 (0.65–0.82)
Physical activity				
Never or once a year	5570 (24.3)	4350 (23.4)	1220 (27.8)	1
Once a month	4147 (18.0)	3421 (18.4)	726 (16.6)	0.77 (0.68–0.88)
Once a week	11046 (48.1)	9012 (48.4)	2034 (46.4)	0.75 (0.67–0.83)
Once a day	2207 (9.6)	1810 (9.7)	397 (9.1)	0.70 (0.59–0.82)

OR, odds ratio; CI, confidence interval.

The variables that were not significant (*p*-value>0.05) on the multivariate level were removed from the table. Sex, age and province were forced in the model.

a Individuals with missing values on one or more variables were excluded from the model. The cultural and racial background variable was excluded from the univariate and multivariate logistic regression because it was highly correlated with immigrant status.

b Based on body mass index international classification for adults ⩾18 years of age.

Nearly one-fifth (19.1%) of our study individuals had immigrated to Canada, the majority >20 years ago (87.5%) and only 1.3% had lived in Canada for <5 years. In multivariate logistic regression models, immigrants (*v.* non-immigrants) were more likely male, older, with post-secondary degree/diploma, to speak English most often at home (*v*. French), unemployed (*v*. employed), with lower incomes, residing in Quebec (*v.* other). Immigrants were less likely single, smokers, living in rural/suburban areas, with bowel disorders or cancer, and less likely overweight or obese (Table 1).

Among immigrants, 12.2% had UD at baseline compared to 10.6% of non-immigrants (Table 2). Risk factors associated with UD at baseline did not differ greatly between immigrants and non-immigrants (online Supplementary Table A). Non-immigrant (but not immigrants) who were unemployed (*v.* employed) or had prior anxiety disorders were at higher risk of UD, while those who exercised at least once a week were at lower risk. Immigrants (but not non-immigrants) who consumed alcohol once a month (*v.* never) and those who were current smokers were at higher risk of UD. Immigrants who arrived in Canada at age >40 years were twice as likely as non-immigrants to have UD (OR 2.02, 95% CI 1.43–2.86). As well, those who resided in Canada for <20 or >40 years were more likely than non-immigrants to have UD (online Supplementary Table B).

**Table 2.** Undiagnosed depression at baseline and depressive symptoms at 18 months by immigration status

Non-immigrants (*N* = 18 620)							
Depression at baseline				Immigrants (*N* = 4382)			
*CES-D 10, N (%)*							
UD (CESD ⩾10)		No-UD (CES-D <10)		UD (CESD ⩾10)		No-UD (CES-D <10)	
1976 (10.6)		16 644 (89.04)		535 (12.2)		3847 (87.8)	
Depressive symptoms at 18 months							
*K10^a^, N (%)*							
⩾19	<19	⩾19	<19	⩾19	<19	⩾19	<19
621 (31.4)	1355 (68.6)	1005 (6.0)	15 639 (94.0)	181 (39.2)	281 (60.8)	231 (8.8)	2407 (91.2)
*Number of respondent to MHCP*^b^							
620	1115	1003	11 142	181	281	231	2407
*Seen an MHCP, N (%)*							
93 (15.0)	75 (6.7)	147 (14.7)	419 (3.8)	31 (17.1)	11 (3.9)	44 (19.1)	82 (3.4)

CES-D, Center for Epidemiological Studies Depression 10 Scale; UD, undiagnosed depression; assessed with Center for Epidemiological Studies Depression 10 Scale, CESD ⩾10; K10, Kessler Psychological Distress Scale 10; MHCP, consulting a mental health care professional for depressive symptoms.

a K10 ⩾19 = depressive symptoms.

b Participants who answered ‘a little’, ‘some’, ‘most’ or ‘all’ to at least one question in the K10_1-10 series were probed about having seen an MHCP about these feelings in the prior 30 days.

In the multivariate logistic regression model evaluating the association between immigrant status and UD, an effect modification of immigrant status by sex was observed. Specifically, among males, immigrant status was not associated with UD (OR 1.05, 95% CI 0.86–1.28), but among females, immigrant status was associated with a 50% increased odd of UD (OR 1.50, 95% CI 1.25–1.80). Female immigrant and female non-immigrant were more likely to be depressed than their male counterparts [immigrant females *v*. immigrant males (OR 1.85, 95% CI 1.45–2.37) and non-immigrant females *v*. non-immigrant males (OR 1.30, 95% CI 1.14–1.47)] (Table 3 and online Supplementary Table C).

**Table 3.** Association between immigrant status and sex and undiagnosed depression at baseline^a^ (*N* = 23 002)

	Unadjusted OR (95% CI)	Adjusted OR (95% CI)
Female immigrant *v.* female non-immigrant	1.46 (1.28–1.75)	1.50 (1.25–1.80)
Female immigrant *v.* male immigrant	2.00 (1.62–2.47)	1.85 (1.45–2.37)
Female non-immigrant *v.* male non-immigrant	1.39 (1.25–1.55)	1.30 (1.14–1.47)
Male immigrant *v.* male non-immigrant	1.04 (0.87–1.25)	1.05 (0.86–1.28)

UD, undiagnosed depression; OR, odds ratio; CI, confidence interval.

a An interaction effect of sex and immigrant was found and is presented here. The multivariate logistic regression models adjusted for all baseline characteristics included in Table 1. The full model is shown in online Supplementary Table C.

Among immigrants with UD at baseline, 34.2% had DS at 18 months, among whom 17.1% had consulted an MHCP in the previous month, while among non-immigrants with UD at baseline, 31.4% had DS at 18 months, among whom 15.0% had consulted an MHCP in the previous month (Table 2). In multivariate logistic regression models, the risk of DS at 18 months was not statistically different between immigrants and non-immigrants whether or not they had UD at baseline. An interaction effect was found between sex and UD at baseline whereby UD increased the risk of DS at 18 months for females (females with UD *v.* females without UD: OR 5.10, 95% CI 4.29–6.06) and for males (males with UD *v.* males without UD: OR 6.02, 95% CI 4.90–7.41), and the risk of UD was higher in females without UD *v.* males without UD, but similar in females with UD *v.* males with UD (Table 4 and online Supplementary Table D).

**Table 4.** Associations of immigrant status with and without undiagnosed depression at baseline with depressive symptoms at 18 months (*N* = 23 002)^a^

	Unadjusted OR (95% CI)	Adjusted OR (95% CI)
Interaction effect of immigrant status and UD at baseline		
Immigrant with UD *v.* immigrant without UD	7.41 (5.73–9.57)	5.37 (4.04–7.14)
Immigrant with UD *v.* non-immigrant with UD	1.08 (0.86–1.37)	1.10 (0.84–1.45)
Immigrant without UD *v.* non-immigrant without UD	1.11 (0.94–1.31)	1.15 (0.95–1.39)
Non-immigrant with UD *v.* non-immigrant without UD	7.56 (6.64–9.62)	5.59 (4.79–6.52)
Interaction effect of sex and UD at baseline^b^		
Female with UD *v.* female without UD	6.71 (5.75–7.81)	5.10 (4.29–6.06)
Female with UD *v.* male with UD	0.95 (0.78–1.15)	1.06 (0.84–1.33)
Female without UD *v.* male without UD	1.21 (1.06–1.37)	1.25 (1.09–1.44)
Male with UD *v.* male without UD	8.47 (7.14–10.20)	6.02 (4.90–7.41)

OR, odds ratio; CI, confidence interval; UD, undiagnosed depression; DS, depressive symptoms.

a The multivariate logistic regression model adjusted for all the variables included in Table 1. The full model is in online Supplementary Table D.

b The model did not show a three-way interaction of immigrant status, sex and UD at baseline.

In multivariate regression models, the overall likelihood of consulting an MHCP at 18 months did not differ between immigrants and non-immigrants (OR 0.95, 95% CI 0.77–1.17) whether or not they had DS. Examining the interaction effect of DS at 18 months and UD at baseline revealed that the likelihood of consulting an MHCP among those with DS did not differ between those with and those without UD at baseline (Table 5 and online Supplementary Table E). Interestingly, those with UD at baseline and no DS (K10 < 19) were 58% more likely to consult an MHCP than those without UD at baseline.

**Table 5.** Associations of immigrant status, baseline undiagnosed depression and depressive symptoms at 18 months with seeing a physician for these feelings in the prior month: multivariate logistic regression models (*N* = 16 519)^a^

	Seeing an MHCP at 18 months	
	Unadjusted OR (95% CI)	Adjusted OR (95% CI)
Immigrant *v.* non-immigrant	1.02 (0.83–1.23)	0.95 (0.77–1.17)
Interaction effect of DS at 18 months and UD at baseline		
DS with UD *v.* no DS with UD	3.01 (2.16–4.18)	3.11 (2.20–4.37)
DS with no UD *v.* no DS with no UD	4.88 (3.99–5.97)	5.05 (4.09–6.24)
DS with UD *v.* DS with no UD	0.93 (0.70–1.23)	0.97 (0.72–1.30)
No DS with UD *v.* no DS with no UD	1.75 (1.34–2.28)	1.58 (1.19–2.09)

CES-D, Center for Epidemiological Studies Depression 10 Scale; K10, Kessler Psychological Distress Scale 10; UD, undiagnosed depression, defined by CES-D score ⩾10 at baseline; DS, depressive symptoms, defined by K10 score ⩾19 at 18 months; MHCP, mental health care professional; OR, odds ratio; CI, confidence interval.

a Multivariate logistic regression models were conducted and the full table can be found in the online Supplementary Table E.

## Discussion

Among 23 002 study participants, one-fifth had immigrated to Canada, and the majority (86%) was over 20 years ago. Female immigrants were more likely to have UD than female non-immigrants, but no difference was observed in men. The risk of UD was higher in immigrants who arrived in Canada at age ⩾40 years and among those who resided in Canada for <20 or >40 years. Persistent DS at 18 months and seeking MHCP for these symptoms did not differ between immigrants and non-immigrants. Of note, only 17% of immigrants and 15% of non-immigrants with persistent DS (DS at 18 months and baseline UD) had consulted an MHCP in the previous month.

As expected, immigrants in our study differed from non-immigrants on all mental health-predisposing, enabling, needs-related and personal health choices considered except for perceived health and alcohol consumption. Similar to other studies, immigrants were more likely to have post-secondary education and lower income (Dunn and Dyck, 2000; Newbold and Danforth, 2003; Setia *et al*., 2012). However, they were less likely to be obese and to be living with pain or cancer (Ali, 2002; Ali *et al.*, 2004; McDonald and Kennedy, 2004; McDonald and Kennedy, 2005; Gushulak *et al*., 2011; Aglipay *et al.*, 2013). Immigrants are reported to be resilient because of their experiences, and hence, probably moderating pain levels (Bauer *et al*., 2016). In terms of cancer and obesity, being an immigrant was seen to be protective in our study. Similar findings were also reported in other Canadian studies among recent immigrants, but over time, the benefits seem to diminish to Canadian norms (McDonald and Kennedy, 2005; Cheung *et al*., 2017).

The risk of UD has not been previously assessed in Canadian immigrants. In a US study, UD was associated with psychosocial stressors including unemployment and relationship problems, but immigration status was not specifically examined (Williams *et al*., 2017). The higher risk of UD found in female immigrants *v.* non-immigrants is in line with the results of other studies that looked at the risk of depression in these groups (Wong and Tsang, 2004; Mechakra-Tahiri *et al.*, 2007; Tang *et al*., 2007; Miszkurka *et al.*, 2010; Guruge *et al*., 2015*b*). The higher exposure to stressors such as post-partum depression, family separation and linguistic, and economic barriers in female immigrants may explain this result (Wong and Tsang, 2004; Mechakra-Tahiri *et al.*, 2007; Tang *et al.*, 2007; Miszkurka *et al*., 2010; George *et al*., 2015; Patten *et al.*, 2015; Guruge *et al.*, 2015*a*). Women are also at higher risk of inflammation and fluctuation of reproductive hormones that make them further susceptible to depression (Yang and Kozloski, 2011). In our study, the risk of UD was similar between male immigrants and non-immigrants. Other Canadian studies also found no association between male sex and depression regardless of immigration status (Stafford *et al*., 2011; Davison *et al.*, 2019).

In our study, immigrants who resided in Canada for <20 years and those who resided for >40 years were at increased risk of UD than the host population. Our findings support a ‘U’ shape association between UD and length of stay in the host country (Beiser, 2005). Immigrants go through several acculturation and integration challenges in their host country during the first 20 years following their migration that might make them vulnerable to anxiety and mood disorders (Berry, 1989; Lay and Nguyen, 1998; Khuwaja *et al*., 2007; Sam and Berry, 2010). These stressors can include economic challenges reflecting the aspects of acceptance by the receiving society, communication barriers, discrimination, loneliness and family structure, lack of social support and cultural adaptation (Mechanic, 1972; Torres *et al*., 2012; Chavez, 2019). Migrants' cultural shaping of symptoms, illness behaviour and coping can delay seeking help (Kirmayer *et al*., 2011) as well as structural healthcare challenges that accommodate ‘cultural distance’ and health inequalities (Saha *et al*., 2008) can delay seeking help. During the following 20–40 years of residence, immigrants then adapt their culturally-defined lifestyles and adopt the norms and behaviours of the host country (Beiser, 2005). However, when residing >40 years in the host country, deterioration in social determinants of health (living alone, lower levels of physical health status, financial status, impaired social integration and social activity) may arise and could explain mood dysfunction at that stage (Kim and Chen, 2011).

Our results also showed an increased risk of UD in those who migrated at ages ⩾40 years. Contrary to our results, one US study reported a lower risk of psychiatric disorders onset in US Latino groups with older ages at arrival (Alegria *et al.*, 2007; Alegria *et al*., 2008). However, other studies reported that Latino immigrants are at higher risk of psychiatric disorders when immigrating during two life cycle periods: before the age of 16 (Vega *et al*., 2004) or after the age of 35 (Mills and Henretta, 2001). Most US studies were conducted in Latino groups which differ from our Canadian cohort who are mostly of South Asian, Black and Chinese backgrounds. Mood disorders and seeking mental health care may differ between ethnic groups, however having a strong community structure of collectivism like in Latino communities might help navigate the healthcare system, and hence, have a positive impact on psychiatric disorders (Ali, 2002; Ali *et al.*, 2004; McDonald and Kennedy, 2004; McDonald and Kennedy, 2005; Gushulak *et al*., 2011; Aglipay *et al.*, 2013). The intricate relationship between UD, age at immigration and residency length requires further clarification (Foo *et al*., 2018).

In our study, immigrants were as likely as non-immigrants to have persistent DS at 18 months and to have consulted an MHCP for these symptoms in the past month. These results differ from those reported by other Canadian studies that found immigrants to be less likely than their Canadian-born counterparts to seek out or be referred to mental health services when they experience comparable levels of distress (Fenta *et al*., 2006; Whitley *et al.*, 2006; Huang *et al.*, 2007). The length of residency (~43 years) in our study may be a possible explanation of the permeability (how easily people can use services) and identification (how need is identified in specific situations) of immigrants in accessing mental health services (Dixon-Woods *et al*., 2006). Immigrants and non-immigrants who had UD at baseline were five times as likely as their counterparts without UD to have DS at 18 months. This highlights the importance of screening and treating depression early to limit the risk of persistent depressive disorders. No other study was found that assessed the likelihood of seeking mental health care among immigrant and non-immigrant with persistent DS.

Strengths of our study include the use of the carefully designed, population-based CLSA database and the high quality of its data. Our study has also some limitations. Although we used the survey weights in our analyses, participation bias cannot be ruled out (Haine *et al*., 2018). Our study included only community-dwelling individuals. As such, vulnerable groups that are particularly at higher risk of depression would be excluded (e.g. homeless, those living in institutions). In addition, the screening tools CES-D at baseline and K10 at 18 months were the only depression-related measures available in the CLSA data at the time of the study. Both CES-D (Cosco *et al*., 2020) and K-10 (Fassaert *et al*., 2009) are reliable and valid instruments to assess DS in the general population. Therefore, we anticipate no changes in our results had the same measurement been available at both time-points. However, CES-D and K10 are based on self-reported information that comes with measuring errors and information bias (Silva Junior *et al*., 2015). Finally, in our study, only information on seeing an MHCP in the past month for their feelings was available.

Future studies should further investigate the personal, cultural and social factors (Dixon-Woods *et al*., 2006) that differentiates newer immigrants (those who reside <20 years) from those who have been in their host country for over 40 years and from the host population as these factors continue to evolve over time with new global challenges and societal structures. It is important to continue assessing the implications of help-seeking factors, cross-cultural differences, social inequalities and other psychological measures over time in large population-based cohorts as with the continued societal changes, cultural barriers and differences of cultural significance of somatic symptoms might need further exploration (Kirmayer *et al*., 2017; Kirmayer *et al.*, 2011). Conducting qualitative work may help gain important insights into our quantitative findings (Kirmayer *et al*., 2011).

To the best of our knowledge, this is the first Canadian study to comprehensively assess associations between UD and immigration status. Screening for depression may particularly benefit female immigrants and those who migrated at 40 years of age and older.

Systematic inquiry into patients' migration trajectory and subsequent follow-up on culturally appropriate indicators of health will allow clinicians to recognise problems in adaptation and undertake mental health promotion, disease prevention or treatment interventions in a timely way. Follow-up screening should query persistence of DS and encourage seeking mental health care regardless of immigration status.

## Data Availability

The authors do not have the permission from the CLSA to share the data. Those who wish to access the data may directly contact the CLSA at email access@clsa-elcv.ca or please visit https://www.clsa-elcv.ca/data-access.

## Supplementary material

For supplementary material accompanying this paper visit http://dx.doi.org/10.1017/S2045796020000670. click here to view supplementary material
