# Supplementary material for: Undiagnosed depression, persistent depressive symptoms and seeking mental health care: analysis of immigrant and non-immigrant participants of the Canadian Longitudinal Study of Aging
Source: Epidemiol Psychiatr Sci. 2020 Aug 14;29:e158. doi: 10.1017/S2045796020000670 (PMC7443777; doi:10.1017/S2045796020000670)
Supplement: Supplementary file 1 [file S2045796020000670sup001.docx]

**Supplemental Material**

Figure 1. Diagram flow chart for sample selection from the Canadian Longitudinal Study on Aging (CLSA).

CES-D = Center for Epidemiological Studies Depression 10 Scale

| **Table A: Risk factors associated with having undiagnosed depression at baseline in immigrant and non-immigrant** | | |
| --- | --- | --- |
|  |  |  |
|  |  |  |
|  | Non-immigrant | Immigrant |
|  | (N=18,620) | (N=4,382) |
|  | Adjusted OR (95% CI) | |
| **Predisposing characteristics** |  |  |
| Age, years, |  |  |
| 45 - 60 | 1 | 1 |
| 61 - 70 | 0.88 (0.74 - 1.05) | 0.87 (0.60 - 1.27) |
| 71 - 85 | 1.04 (0.85 - 1.28) | 0.85 (0.55 - 1.29) |
| Sex |  |  |
| Male | 1 | 1 |
| Female | 1.29 (1.13 - 1.47) | 2.06 (1.56 - 2.71) |
| Marital status |  |  |
| Single | 1 | 1 |
| Married | 0.78 (0.63 - 0.97) | 0.47 (0.30 - 0.75) |
| Widowed/divorced/separated | 0.97 (0.78 - 1.21) | 0.54 (0.33 - 0.89) |
| Cultural and racial background |  |  |
| White |  | 1 |
| Black | 0.74 (0.20 - 2.75) | 0.76 (0.39 - 1.47) |
| South Asian | 3.63 (0.87 - 15.15) | 3.33 (2.09 - 5.29) |
| Chinese | 1.53 (0.53 - 4.45) | 0.89 (0.43 - 1.84) |
| First Nations | 1.17 (0.49 - 2.78) | - |
| Other | 1.23 (0.55 - 2.75) | 1.28 (0.79 - 2.08) |
| Length of residence in Canada (years) |  |  |
| 0-5 | - | 1 |
| 6-10 |  | 0.56 (0.20 - 1.54) |
| 11-20 | - | 0.46 (0.18 - 1.18) |
| 21-40 | - | 0.34 (0.14 - 0.82) |
| > 40 | - | 0.44 (0.18 - 1.07) |
| **Enabling resources** |  |  |
| Total household income Can $ |  |  |
| < 20,000 | 1 | 1 |
| 20,000- less than 50,000 | 0.90 (0.69 - 1.19) | 0.44 (0.24 - 0.81) |
| 50,000- less than 100,000 | 0.72 (0.54 - 0.95) | 0.36 (0.19 - 0.67) |
| ≥ 100,000 | 0.54 (0.40 - 0.74) | 0.28 (0.14 - 0.56) |
| Working status |  |  |
| Employed | 1 |  |
| Unemployed | 1.67 (1.28 - 2.19) | 1.34 (0.75 - 2.40) |
| Retired | 0.86 (0.72 - 1.02) | 0.97 (0.68 - 1.38) |
| Province |  |  |
| Quebec | 1 | 1 |
| British Columbia | 0.80 (0.65 - 0.98) | 0.87 (0.59 - 1.29) |
| Ontario | 0.94 (0.78 - 1.15) | 0.89 (0.59 - 1.32) |
| Other | 1.02 (0.87 - 1.21) | 0.95 (0.65 - 1.40) |
| **Needs-related factors** |  |  |
| Perceived Health |  |  |
| Poor | 1 | 1 |
| Fair | 0.89 (0.51 - 1.56) | 1.57 (0.55 - 4.44) |
| Good | 0.43 (0.25 - 0.75) | 0.72 (0.27 - 1.93) |
| Very Good | 0.26 (0.15 - 0.44) | 0.44 (0.16 - 1.19) |
| Excellent | 0.16 (0.09 - 0.28) | 0.22 (0.08 - 0.63) |
| Medical Conditions (Yes vs. No) |  |  |
| Living with pain | 1.71 (1.50 - 1.95) | 1.71 (1.32 - 2.21) |
| Bowel disorders | 1.32 (1.08 - 1.60) | 1.29 (0.78 - 2.14) |
| Arthritis | 0.60 (0.42 - 0.86) | 1.24 (0.60 - 2.55) |
| Anxiety disorder | 2.63 (2.04 - 3.39) | 1.46 (0.74 - 2.89) |
| **Personal health choices** |  |  |
| Alcohol consumption |  |  |
| Never | 1 | 1 |
| About once a month | 0.80 (0.64 - 1.00) | 1.60 (1.02 - 2.52) |
| 2-4 times a month | 0.80 (0.65 - 0.99) | 1.53 (0.95 - 2.45) |
| >2 times a week | 0.83 (0.68 - 1.01) | 1.23 (0.78 - 1.95) |
| Smoking status |  |  |
| Smoker | 1 | 1 |
| Former smoker | 0.92 (0.75 - 1.13) | 0.58 (0.37 - 0.92) |
| Non-smoker | 0.83 (0.66 - 1.04) | 0.44 (0.27 - 0.71) |
| Weight classification ^a^ |  |  |
| Normal weight | 1 | 1 |
| Underweight | 0.87 (0.46 - 1.64) | 0.10 (0.01 - 0.97) |
| Overweight | 0.87 (0.74 - 1.01) | 0.86 (0.64 - 1.17) |
| Obese | 0.81 (0.69 - 0.95) | 0.68 (0.48 - 0.97) |
| Physical activity |  |  |
| Never or once a year | 1 | 1 |
| Once a month | 0.94 (0.79 - 1.13) | 1.03 (0.70 - 1.52) |
| Once a week | 0.75 (0.64 - 0.87) | 0.97 (0.71 - 1.32) |
| Once a day | 0.68 (0.53 - 0.88) | 0.95 (0.57 - 1.56) |
| OR=Odds Ratio; CI=Confidence Intervals  The multivariate logistic regression model adjusted for all baseline characteristics included in Table 1. Variables that were not significant on the multivariate level were removed from the table. Sex, age and province were forced in the model.  ^a^ Based on Body Mass Index international classification for adults aged 18 and over. | | |
|  |  |  |

| **Table B: Association of age of immigrant at arrival and length of residence in Canada with undiagnosed depression at baseline: multivariate logistic regression models (N=23,002)** | | |
| --- | --- | --- |
|  |  |  |
|  | Undiagnosed depression ^a^ versus not | |
|  | Unadjusted OR (95% CI) | Adjusted OR (95% CI) |
| Model 1 ^b^  Age at arrival in Canada (years old) |  |  |
| Non-immigrant | 1 | 1 |
| 0-5 | 1.07 (0.82 - 1.40) | 1.20 (0.89 - 1.60) |
| 6-17 | 1.09 (0.84 - 1.41) | 1.21 (0.89 - 1.63) |
| 18-22 | 1.32 (1.00 - 1.74) | 1.18 (0.86 - 1.63) |
| 22-40 | 1.16 (0.98 - 1.37) | 1.17 (0.95 - 1.42) |
| > 40 | 2.21 (1.64 - 2.97) | 2.02 (1.43 - 2.86) |
| Model 2 ^b^  Length of residence in Canada (years) |  |  |
| Non-immigrant | 1 | 1 |
| 0-5 | 4.47 (2.45 - 8.17) | 3.30 (1.59 - 6.85) |
| 6-10 | 1.79 (1.10 - 2.91) | 1.84 (1.08 - 3.11) |
| 11-20 | 1.39 (0.98 - 1.96) | 1.53 (1.02 - 2.29) |
| 21-40 | 1.10 (0.88 - 1.36) | 1.03 (0.80 - 1.33) |
| > 40 | 1.15 (1.00 - 1.33) | 1.21 (1.02 - 1.43) |

OR=Odds Ratio; CI=Confidence Interval;

^a^ Undiagnosed depression at baseline was assessed using Center for Epidemiological Studies Depression 10 (CES-D10) Scale ≥10;

^b^ Both Model 1 and 2 included age, sex, province, income, marital status, smoking, perceived health, anxiety disorder, living with pain, bowel disorders, weight, physical activity. Note that variables that were not significant on the univariate level were removed from the model.

| **Table C: Association of immigrant status and sex with undiagnosed depression at baseline**^a^**: multivariate logistic regression models (N=23,002)** | |
| --- | --- |
|  |  |
|  |  |
|  |  |
|  | Adjusted OR (95% CI) |
| Female immigrant versus female non-immigrant | 1.50 (1.25 - 1.80) |
| Female immigrant versus male immigrant | 1.85 (1.45 - 2.37) |
| Female non-immigrant versus male non-immigrant | 1.30 (1.14 - 1.47) |
| Male immigrant versus male non-immigrant | 1.05 (0.86 - 1.28) |
| **Predisposing characteristics** |  |
| Age, y |  |
| 45-60 | 1 |
| 61-70 | 0.76 (0.66 - 0.86) |
| 71-85 | 0.84 (0.73 - 0.96) |
| Marital Status |  |
| Single | 1 |
| Married | 0.75 (0.62 - 0.91) |
| Widowed/Divorced/Separated | 0.92 (0.75 - 1.11) |
| **Enabling resources** |  |
| Total household income Can $ |  |
| < 20,000 | 1 |
| 20,000- less than 50,000 | 0.72 (0.57 - 0.92) |
| 50,000- less than 100,000 | 0.58 (0.45 - 0.74) |
| ≥100,000 | 0.43 (0.33 - 0.56) |
| Province |  |
| Quebec | 1 |
| British Columbia | 0.86 (0.72 - 1.02) |
| Ontario | 0.95 (0.80 - 1.23) |
| Other | 1.04 (0.90 - 1.21) |
| **Needs-related factors** |  |
| Perceived Health |  |
| Poor | 1 |
| Fair | 0.95 (0.59 - 1.51) |
| Good | 0.47 (0.30 - 0.75) |
| Very Good | 0.27 (0.17 - 0.43) |
| Excellent | 0.16 (0.10 - 0.26) |
| Medical Conditions (Yes vs. No) |  |
| Living with pain | 1.64 (1.47 - 1.84) |
| Bowel Disorders | 1.29 (1.08 - 1.54) |
| Anxiety disorder | 2.34 (1.85 - 2.95) |
| **Personal health choices** |  |
| Smoking status |  |
| Smoker | 1 |
| Former smoker | 0.85 (0.70 - 1.02) |
| Non-smoker | 0.75 (0.61 - 0.91) |
| Weight classification ^b^ |  |
| Normal weight | 1 |
| Underweight | 0.70 (0.38 - 1.29) |
| Overweight | 0.85 (0.75 - 0.97) |
| Obese | 0.78 (0.68 - 0.90) |
| Physical activity |  |
| Never or once a year | 1 |
| Once a month | 0.93 (0.79 - 1.09) |
| Once a week | 0.75 (0.66 - 0.86) |
| Once a day | 0.70 (0.56 - 0.87) |
| OR = Odds ratio, CI = Confidence Interval;  ^a^ Undiagnosed depression at baseline was assessed using Center for Epidemiological Studies Depression 10 (CES-D10) Scale ≥10;  ^b^ Based on Body Mass Index international classification for adults ≥ 18 years of age. | |
|  |  |

| **Table D: Associations of immigrant status with and without undiagnosed depression at baseline with depressive symptoms at 18 months: multivariate logistic regression models (N=23,002)** | |
| --- | --- |
|  |  |
|  |  |
|  | Adjusted OR (95% CI) |
| Interaction effect of Immigrant status and UD at baseline |  |
| Immigrant with UD versus immigrant without UD | 5.37 (4.04 - 7.14) |
| Immigrant with UD versus non-immigrant with UD | 1.10 (0.84 - 1.45) |
| Immigrant without UD versus non-immigrant without UD | 1.15 (0.95 - 1.39) |
| Non-immigrant with UD versus non-immigrant without UD | 5.59 (4.79 - 6.52) |
| Interaction effect of sex and UD at baseline ^a^ |  |
| Female with UD versus female without UD | 5.10 (4.29 - 6.06) |
| Female with UD versus male with UD | 1.06 (0.84 - 1.33) |
| Female without UD versus male without UD | 1.25 (1.09 - 1.44) |
| Male with UD versus male without UD | 6.02 (4.90 - 7.41) |
| **Predisposing characteristics** |  |
| Age, years, |  |
| 45 - 60 | 1 |
| 61 - 70 | 0.77 (0.66 - 0.91) |
| 71 - 85 | 0.77 (0.64 - 0.93) |
| Cultural and racial background |  |
| White | 1 |
| Black | 0.91 (0.50 - 1.64) |
| South Asian | 1.49 (0.91 - 2.44) |
| Chinese | 1.62 (0.91 - 2.89) |
| Other | 1.57 (1.10 - 2.25) |
| **Enabling resources** |  |
| Total household income Can $ |  |
| < 20,000 | 1 |
| 20,000- less than 50,000 | 0.68 (0.53 - 0.88) |
| 50,000- less than 100,000 | 0.60 (0.46 - 0.77) |
| ≥100,000 | 0.44 (0.34 - 0.58) |
| Working status |  |
| Employed | 1 |
| Unemployed | 1.16 (0.88 - 1.52) |
| Retired | 0.69 (0.59 - 0.81) |
| Province |  |
| Quebec | 1 |
| British Columbia | 0.51 (0.42-0.61) |
| Ontario | 0.55 (0.46-0.65) |
| Other | 0.52 (0.45-0.60) |
| **Needs-related factors** |  |
| Perceived Health |  |
| Poor | 1 |
| Fair | 0.64 (0.39 - 1.06) |
| Good | 0.47 (0.29 - 0.77) |
| Very Good | 0.30 (0.18 - 0.48) |
| Excellent | 0.21 (0.13 - 0.36) |
| Medical Conditions |  |
| Living with pain | 1.59 (1.41 - 1.80) |
| Anxiety disorder | 2.21 (1.73 - 2.84) |
| **Personal health choices** |  |
| Smoking status |  |
| Smoker | 1 |
| Former smoker | 0.69 (0.57-0.83) |
| Non-smoker | 0.69 (0.57-0.83) |
| OR=Odds Ratio; CI=Confidence Interval; UD=Undiagnosed Depression  Undiagnosed depression at baseline is assessed using Center for Epidemiological Studies Depression 10 (CES-D10) Scale ≥10; | |
| ^a^ The model adjusted for all the variables included in Table 1. | |

| **Table E: Associations of immigrant status, baseline undiagnosed depression and depressive symptoms at 18 months with seeing a physician for these feelings in the prior month: multivariate logistic regression models (N=16,519)**   \|  \| Seeing a mental health care professional at 18 months \| \| \| --- \| --- \| --- \| \| Unadjusted OR (95% CI) \| Adjusted OR (95% CI) \| \| Immigrant versus non-immigrant \| 1.02 (0.83 - 1.23) \| 0.95 (0.77 - 1.17) \| \| UD with DS versus UD with no DS \| 3.01 (2.16 - 4.18) \| 3.11 (2.20 - 4.37) \| \| UD with DS versus no UD with DS \| 0.93 (0.70 - 1.23) \| 0.97 (0.72 - 1.30) \| \| No UD with DS versus no UD with no DS \| 4.88 (3.99 - 5.97) \| 5.05 (4.09 - 6.24) \| \| UD with no DS versus no UD and no DS \| 1.75 (1.34 - 2.28) \| 1.58 (1.19 - 2.09) \| \| **Predisposing characteristics** \|  \|  \| \| Age, y \|  \|  \| \| 45 - 60 \| 1 \| 1 \| \| 61 - 70 \| 0.85 (0.71 - 1.01) \| 0.88 (0.73 - 1.05) \| \| 71 - 85 \| 1.05 (0.88 - 1.25) \| 0.98 (0.81 - 1.18) \| \| Sex \|  \|  \| \| Male \| 1 \| 1 \| \| Female \| 1.35 (1.16 - 1.58) \| 1.22 (1.03 - 1.43) \| \| **Enabling resources** \|  \|  \| \| Province \|  \|  \| \| Quebec \| 1 \| 1 \| \| British Columbia \| 1.21 (0.94 - 1.57) \| 1.54 (1.18 - 2.02) \| \| Ontario \| 1.50 (1.17 - 1.93) \| 1.82 (1.41 - 2.35) \| \| Other \| 1.68 (1.35 - 2.10) \| 1.97 (1.56 - 2.48) \| \| **Needs-related factors** \|  \|  \| \| Medical Conditions \|  \|  \| \| Living with pain \| 1.72 (1.48 - 2.01) \| 1.38 (1.17 - 1.63) \| \| Bowel disorders \| 1.81 (1.43 - 2.29) \| 1.45 (1.12 - 1.88) \| |
| --- | --- | --- | --- | --- | --- | --- | --- | --- | --- | --- | --- | --- | --- | --- | --- | --- | --- | --- | --- | --- | --- | --- | --- | --- | --- | --- | --- | --- | --- | --- | --- | --- | --- | --- | --- | --- | --- | --- | --- | --- | --- | --- | --- | --- | --- | --- | --- | --- | --- | --- | --- | --- | --- | --- | --- | --- | --- | --- | --- | --- | --- | --- | --- | --- | --- | --- | --- | --- | --- | --- | --- | --- | --- | --- |
|  |
| CES-D=Center for Epidemiological Studies Depression 10 Scale; K10=Kessler Psychological Distress Scale 10; UD=Undiagnosed Depression, defined by CES-D score ≥ 10 at baseline; DS=Important Depressive Symptoms, defined by K10 score ≥ 19 at 18 months; OR=Odds Ratio; CI=Confidence Interval;  Note that variables that were not significant on the univariate level were removed from the table. Sex, age and province were forced in the model. |
